# Role of interferon-induced GTPases in leishmaniasis

**DOI:** 10.1371/journal.pntd.0010093

**Published:** 2022-01-27

**Authors:** Marie Lipoldová, Yahya Sohrabi

**Affiliations:** 1 Institute of Molecular Genetics of the Czech Academy of Sciences, Prague, Czech Republic; 2 University Hospital Münster, Münster, Germany; University of São Paulo FMRP/USP, BRAZIL

Leishmaniasis continues to be a major health problem worldwide. The efficacy of reported vaccines is poor, and there is no safe and cost-effective treatment, partly because the mechanisms of the disease are not fully understood. Recent studies have shed light on the role of interferon (IFN)-inducible GTPases as regulators of immunity to infection. Farias Amorim and colleagues [1] describe differences in the transcriptome of blood cells isolated from patients infected with *Leishmania braziliensis* and healthy controls. They highlighted IFNγ and cytolytic transcriptional signatures characteristic for this cutaneous leishmaniasis. Their findings included overexpression of genes encoding guanylate-binding proteins (GBPs): *GBP1*, *GBP2*, *GBP3*, *GBP4*, *GBP5*, and *GBP6* and the pseudogene *GBP1P1*. All detected GBPs except *GBP2* were positively correlated with cell type abundance scores for monocytes and macrophage/monocyte-like cells using the microenvironment cell population counter. However, the complexity of GBPs involvement in anti-*Leishmania* responses deserves additional attention.

## GBPs are components of cell-autonomous immunity against intracellular pathogens

In anti-infection response, GBPs interact with various host pathways and proteins mediating pathogen control via multiple mechanisms such as inflammasome activation, destabilization of pathogen compartments and membranes, destruction of pathogen via autophagy, recruitment of NADPH oxidases with subsequent production of reactive oxygen species, and inhibition of pathogen mobility [2,3]. GBPs play an important role in response to multiple viral, bacterial, and protozoan pathogens [2–4], both vacuolar [2,5] and cytosolic [5]. Many GBPs are expressed constitutively; increase of their expression was shown to be induced by inflammation triggers such as IFNγ, IFNα, IFN-λs1-3, tumor necrosis factor alpha (TNFα), and interleukin (IL)-1α/β in a range of cell types, including B cells and T cells, fibroblasts, endothelial cells, keratinocytes, monocytes, and macrophages [3]. Functional studies and experiments with genetically engineered mouse models showed that 2 of GBPs inducers, IFNγ and TNFα, are important factors in defense against *Leishmania* parasites [6]. Extensive analyses of host–pathogen interactions revealed multiple molecular effectors mediating effects of IFNγ on pathogens by direct killing, production of killing molecules, or limiting pathogen growth [7]. Some of these molecular effectors such as NADPH oxidase (Phox), nitric oxide synthase (NOS2), indoleamine 2,3-dioxygenase (IDO) [8], and natural resistance–associated macrophage protein 1/solute carrier family 11 member 1 (NRAMP1/SLC11A1) [9] have been shown to participate in defense against *Leishmania* parasites. There are several intriguing questions: Could GBPs serve as one of IFNγ effectors, or could they operate independently of the cytokine network? Does their involvement in defense pathways depend on *Leishmania* species? Does the response mediated by GBPs depend on the cell type? And lastly, does the antimicrobial defense mediated by GBPs depend on the stage of the disease?

## Increase of GBPs after *Leishmania* infection

Infection of bone marrow–derived macrophages from BALB/c mice with *Leishmania major* promastigotes led to up-regulation of expression of *Gbp2b/Gbp1*, *Gbp2*, *Gbp3*, *Gbp6*, and *Gbp7* mRNA [10]. Infection of mouse embryonic fibroblasts (MEFs) and peritoneal exudate cells (PECs) derived from the strain C57BL/6 with *Leishmania donovani* led to increase of *Gbp2* mRNA and GBP2 protein [11]. Dendritic cells generated from blood of healthy human donors infected with *L*. *major* promastigotes expressed higher levels of both *GBP1* and *GBP2* mRNA [12], whereas only *GBP1* had increased expression in dendritic cells [12] and human alveolar basal epithelial adenocarcinoma (A549) cells [11] infected with *L*. *donovani*. Up-regulation of *GBP5* mRNA was observed in skin lesions of *L*. *braziliensis*–infected patients [13], whereas blood cells displayed increased expression of *GBP1* to *GBP6* transcripts, with *GBP5* and *GBP1* exhibiting the highest up-regulation [1].

## Role of *GBP2b* in host defense to *Leishmania* infections

Previous data indicate that expression of *Gbp2b* and *Gbp5* mRNA were elevated in skin, inguinal lymph nodes, spleen, and liver tissue of resistant, intermediate, and susceptible mouse strains in the chronic phase of *L*. *major* infection [14]. The increased expression of these genes was more pronounced in skin. Each of the 10 tested strains, including the resistant strains, exhibited increased expression of *Gbp2b* and/or *Gbp5* mRNA in at least 1 organ after infection. The data indicate that *Gbps* expression is tissue specific and highly regulated by host genetic background. There was a tight colocalization of GBP2 protein with most *L*. *major* parasites in skin of resistant and intermediate strains STS, CcS-5, O20, and CcS-20, whereas in a highly susceptible strain BALB/c, most parasites did not associate with GBP2. This suggests a role of GBP2 in the defense against leishmaniasis [14].

## Direct evidence that GBPs participate in *Leishmania* killing *in vitro*

Experiments using nonphagocytic cells provided direct evidence of the killing of *L*. *donovani* [11]. Generally, *Leishmania* parasites reside in *Leishmania*-containing vacuoles (LCVs) and develop a range of reactions to block the fusion of these LCVs with lysosomes. Monitoring MEFs and A549 cells revealed that mouse GBPs and human GBP1 do not efficiently target LCVs in MEFs and A549 cells, respectively, but facilitate the recruitment of lysosomal markers like lysosome-associated membrane proteins (LAMPs) and the autophagosome marker light chain 3 (LC3) to the LCVs. This promotes parasites clearance via autophagy [11]. Interestingly, the pretreatment with recombinant IFNγ did not have any additional leishmanicidal effect. The authors propose that this GBP-dependent host defense program makes nonphagocytic cells an inhospitable host for *Leishmania* growth [11].

## GBPs mediate innate immunity

Recent studies have revealed that GBPs play an important role in controlling inflammation and innate immune functions, in fact even beyond cell-autonomous immune responses. GBPs mediate inflammasome activation that promotes maturation of pro-inflammatory cytokines IL-1β and IL-18 in response to microbial triggers [4]. To assemble the inflammasome, various pattern and damage-associated molecular patterns (PAMPs and DAMPs) provide the activation signal. Harrington and Gurung have shown a controversial role of inflammasome activation in murine models of leishmaniasis [15]. Interestingly, these studies suggest that a protective role for inflammasome against *Leishmania* infection are performed with C57BL/6 background mice, whereas the studies showing a pathological role of inflammasome activation in leishmaniasis have been conducted in susceptible BALB/c mice, indicating considerable influence of the genotype on the outcome [15]. Given the role of GBPs in inflammasome assembly, these data confirm the influence of the genetic background on the protective role of GBPs in leishmaniasis [14]. The role of GBPs in inflammasome activation is not only important in immune responses against infection, but could also be particularly important in immune priming and vaccine development.

## Conclusions

Expression of *mGbps* and/or *hGBPs* is increased in different cell types and organs after infection with at least 3 *Leishmania* species: *L*. *braziliensis* [1,13], *L*. *donovani* [11], and *L*. *major* [10,12,14]. GBPs are involved in killing *L*. *donovani* in nonphagocytic cells [11] and might be involved in control of *L*. *major* in skin [14]. It remains to be elucidated whether expression of GBPs increases after infection with other *Leishmania* spp. and what is their role in the main *Leishmania* host cells—professional phagocytes.
